# ALIVIADO HOME HEALTH AND HOSPICE AIDE DEMENTIA CARE EXPERT PROGRAM IMPROVES AIDE DEMENTIA KNOWLEDGE

**DOI:** 10.1093/geroni/igac059.339

**Published:** 2022-12-20

**Authors:** Shih-Yin Lin, Aditi Durga, Ariel Ford, S Raquel Ramos, Michele Crespo-Fierro, Tina Sadarangani, Abraham Brody

**Affiliations:** New York University, New York, New York, United States; New York University, New York, New York, United States; New York University Rory Meyers College of Nursing, New York, New York, United States; New York University Rory Meyers College of Nursing, New York, New York, United States; New York Univeresity Rory Meyers College of Nursing, New York, New York, United States; New York University, New York, New York, United States; HIGN at the NYU Rory Meyers College of Nursing, New York, New York, United States

## Abstract

Most persons living with dementia receive home health or hospice aide services during their hospice stay. To equip aides with essential knowledge and skills to effectively communicate with and care for persons living with dementia, we developed a 17-video dementia care expert program for aides, available in both English and Spanish. The objective of this presentation is two-fold: first, describe the development and refinement of the Aliviado aide dementia care expert program, including topic selection, usability testing, and cultural tailoring; and second, discuss aide knowledge improvement, along with special considerations for training aides during COVID-19. The Aliviado dementia care expert program for aides has been completed by 414 aides across 15 hospices as of February 2022; of which, 384 aides (93%) completed a dementia knowledge assessment both before and after the training and demonstrated significant knowledge gains with moderate-to-large effect sizes (p<0.0001; Cohen’s d=0.63).

